# The use of non-prescription medicine versus self-assessed health: evidence from Malawi

**DOI:** 10.1186/1755-7682-4-38

**Published:** 2011-11-24

**Authors:** Jacob Novignon, Richard Mussa, Tinkhani Msonda, Justice Nonvignon

**Affiliations:** 1 Department of Economics, University of Malawi, Zomba, Malawi; 2 United Nations Office, Lilongwe, Malawi; 3 Department of Health Policy, Planning & Management, School of Public Health, University of Ghana, Legon, Ghana

**Keywords:** Non-prescription medicine, health status, self-assessed health, ordered probit model, Malawi

## Abstract

**Background:**

The use of medicine is an important part of any health care process and the improvement of health status of any population. While some medicines are legitimately prescribed by practitioners, others take drugs not prescribed by practitioners when they suffer from illness or injuries. The effect of such actions on the health of individuals cannot be overlooked. Even though majority of health policies in developing countries have focused on chronic diseases and the functioning of health systems, abuse of drugs or medicines is a serious population health problem that deserves equal attention. The objectives of the current study are to examine the social and economic dimensions of the use of non-prescription medicines and to determine the effect it has on self-assessed health of individuals.

**Methods:**

The study employs data from the second Integrated Household Survey of Malawi with a sample of 2506 individuals who reported an incidence of illness or injury over the past two weeks before the survey. Regression analysis is conducted at two levels, first is a probit model to identify socio-economic factors that influence the use of non-prescription medicine. The second step uses an ordered probit to model the effect of the use of non-prescription medicines on self-assessed health of individuals.

**Results:**

Results from the probit model show that availability of a health facility in the community negatively affects the use of non-prescription medicines. Age of the individual and the total household health expenditure relate to higher use of non-prescription medicine. Results from the ordered probit model shows that individuals who used non-prescription medicines were likely to report lower categories of self-assessed health.

**Conclusions:**

While policy makers need to engage in public campaign to educate the population on the health risks posed by the use of non-prescription medicines, attention also has to be paid to the social and economic characteristics of the population. Efforts to provide health facilities in communities where they do not exist and improve existing ones will be a crucial step in reducing the use of non-prescription medicines.

## Background

According to the World Health Organization (WHO), rational medicine use requires that "a patient receive medications appropriate to their clinical needs, in doses that meet their own individual requirements, for an adequate period of time, and at the lowest cost to them and their community"[1]. It is estimated that more than 50% of all medicines are prescribed, dispensed or sold inappropriately and that half of all patients are unable to take these medicines correctly [1]. Medical prescriptions may differ across individuals under various conditions. For instance, some medicines are taken with meals or after meals and others not suitable for pregnant women or children under specific ages. Moreover, while some medicines may have mild or unnoticeable side effects, others have side effects that require medical attention. Neilsen et al [2] emphasise that there is a significant relationship between the use of medicine and health status.

Non-prescription medicine, in this context, refers to medicine purchased from a drug/grocery store and used without prescription from a qualified practitioner. In Malawi, non-prescription medicines include antimalarials, antibiotics, pain killers and cough syrups. As is the case in many sub-Saharan African countries, these medicines could be purchased from chemical shops that are available in almost every small town and there is no policy restricting the purchase of these medicines from chemical stores. Such medicines are likely to be overused, underused or misused, which may result in widespread health hazards [1].

The use of non-prescription medicine is associated with various social and economic factors such as sex, income, education and health status [3,4]. Nonvignon et al. [5] show that factors that could encourage self-medication include longer travel and waiting times at public health facilities. Non-prescription medicines also influence health status as perceived by an individual [6,7]. It is worth noting that perceived or self-assessed health has been shown to relate positively with health status and mortality [8-11]. Fillenbaum et al [12] also found that poorer self-assessed health encourages use of non-prescription medicine.

The purpose of this study is to examine the factors that influence the use of non-prescription medicines and assess if and how non-prescription medicine use influences self-assessed health in Malawi.

### Brief country profile

Malawi is a low income country located in southern Africa with an estimated population of 13,077,160 as at 2008 [13]. Gross domestic product (GDP) is estimated to be US$2,920 million in 2008 figures with annual GDP growth rate of 7.6% and per capita gross national income (GNI) of US$289.0 [14]. Total health expenditure as a percentage of GDP was 9.9% in 2007, with government expenditure being 59.7% and private sector expenditure being 40.3% [15] of total health expenditure.

Table 1 show that Malawi has a physician density per 10,000 population of less than 0.5 compared to an average of 2 for the African region. Further, density of nurses and midwives per 10,000 population is 3 in Malawi and 11 (average) in the African region [15]. The density of pharmacists per 10,000 population is less than 0.5 in Malawi and an average of 1 in the Africa region. In Malawi, density of hospital beds per 10,000 population is estimated to be 11 while the entire African region has an estimate of 9 (Table 1).

**Table 1:** Health workforce and infrastructure, 2009

	Malawi		Africa Region	
**Health workforce**	**Number**	**Density per 10000 population**	**Number**	**Density per 10000 population**

Physicians	257	< 0.5	174510	2
Nursing and Midwifery	3896	3	802076	11
Dentistry	211	< 0.5	25798	< 0.5
Pharmacists	293	< 0.5	56212	1
Public Health Workers	318	< 0.5	28856	< 0.5
Community Health Workers	10055	7		
Hospital Beds		11		9

Source: WHO (2010)

## Methods

### Data

The study uses cross section data from the second Integrated Household Survey (IHS2) which was conducted by the National Statistics Office (NSO) of Malawi in 2004-2005 [16]. The survey sample was drawn using a two-stage stratified sampling procedure [17]. The sample frame includes all the three (3) regions of Malawi (i.e. North, South and Central regions) with stratifications on the basis of urban and rural strata. The urban stratum includes all the four major urban areas (i.e. Lilongwe, Blantyre, Mzuzu and Zomba). All other areas were considered to be rural [17].

The data covered issues of household behaviour and welfare, distribution of income, employment, health and education. The total number of respondents was 11,280 households. Out of this number, 2,506 households reported an incidence of illness or injury over the two weeks preceding the survey [17] and this sample was used in this study.

The survey collected information on the use of non-prescription medicine by asking if household member used such medicines and how much was spent on them. Examples of non-prescription medicines mentioned in the questionnaire are Fancida, Panadol and Cough syrups. The survey also asked respondents to describe their current health status compared to what it was a year earlier by choosing an item on a five-point scale (i.e. much better, somewhat better, about the same, somewhat worse and much worse).

### Estimation process

The study employs two models. First, a probit model was used to examine the effects of socio-economic factors on the use of non-prescription medicine. Second, an ordered probit model was used to assess the relationship that exists between the use of non-prescription medicine and self-assessed health after controlling for potential confounders.

### The probit model

Following Jones [18], the model assumes that there is some unobservable continuous latent variable V_i_ * that determines the use of non-prescription medicine during an illness or injury,

V_i _= 1 if and only if V_i_* > 0,

and

V_i _= 0 if and only if V_i_* ≤ 0

The natural regression model for V_i_* can be in the form of index function models so that, the latent variable (V_i_*) is modelled as a linear regression function of the individuals' characteristics (X) [19];

$$V_{i}*=X_{i}\beta +\varepsilon_{i}$$

Where β is a vector of coefficients and ε is the error term which has a standard normal distribution with zero mean and constant variance.

Since the latent variable cannot be observed, the probability (p) of an individual using non-prescription medicine is computed as

$$\begin{array}{ll} p\left(V_{i}=1∕X_{i}\right) & =P\left(V_{i}*>0∕X_{i}\right)\;\\ & =p\left(X_{i}\beta +\varepsilon_{i}>0\right)\;\\ & =p\left(\varepsilon_{i}>-X_{i}\beta \right)\;\\ & \; \end{array}$$

The estimations are obtained by maximizing the loglikelihood function

LogL=∑i(1-yi)log(1-F(xiβ))+yi log(F(xiβ))

### The ordered probit model

Following Greene [19], the starting point of the model is formulation of a latent variable H* that is unobserved i.e. an individual's "true" health which depends on a linear combination of explanatory variables:

$$H*=\beta^{\prime }x+\overset{\prime }{\varepsilon }$$

Where *x *is a set of explanatory variables, β is a set of coefficients and έ is an error term assumed to be uncorrelated with the set of regressors.

What can, however, be observed is the following:

*H_i _*= 1 if H* ≤ 1 e.g much better

*H_i _*= 2 if 1 ≤ H* ≤ *α*_1 _e.g somewhat better

*H_i _*= 3 if *α*_1 _<H* ≤ *α*_2 _e.g about the same.

*H_i _= J *if *α_J_*_-1_<H*

The α's are unknown parameters to be estimated with the β. Notice that *α*_0 _= -∞ and *α_J _*= ∞

The probability of observing *H_i _*= *j *equals the probability that the estimated linear function is within the cut-off points estimated for the outcome

$$\begin{array}{l} P_{ij}=\mathrm{Pr}\left[H_{i}=j\right]=\\ \mathrm{Pr}\left[\alpha_{j-1}<\left(\beta_{1}x_{1i}+\beta_{2}x_{2i}+...+u_{i}\right)\leq \alpha_{j}\right] \end{array}$$

In the ordered probit model έ is assumed to be normally distributed across observations with mean and variance normalized to 0 and 1 respectively.

The probabilities of each category are:

$$\mathrm{Pr}ob\left(H_{i}=0|x_{i}\right)=\text{Φ}\left(-x_{i}\beta \right)$$

$$\mathrm{Pr}ob\left(H_{i}=1|x_{i}\right)=\text{Φ}\left(\alpha_{1}-x_{i}\beta \right)-\text{Φ}\left(-x_{i}\beta \right)$$

$$\mathrm{Pr}ob\left(H_{i}=J|x\right)=1-\text{Φ}\left(\alpha_{J-1}-x_{i}\beta \right)$$

The function Φ (.) denotes the standard normal distribution. The corresponding estimators are obtained by maximizing the log-likelihood function:

$$\begin{array}{l} \text{Γ}\left(\beta ,\alpha \right)=\underset{Y=0}{\sum }\log\left[\mathrm{Pr}\left(H_{i}=0|X_{i}\right)\right]+\\ \underset{Y=1}{\sum }\log\left[\mathrm{Pr}\left(H_{i}=1|X_{i}\right)\right]+...+\underset{Y=J-1}{\sum }\log\left[\mathrm{Pr}\left(H_{i}=\left(J-1|X_{i}\right)\right)\right] \end{array}$$

The signs of the coefficients show the tendency of the variation in the probability of belonging to the highest answer due to an increase in the corresponding explanatory variable. A negative coefficient means that an increase in the independent variable has the effect of increasing the probability of being in a higher category of the dependent variable [19,20].

## Results

### Descriptive statistics

Table 2 shows that 56% of respondents used non-prescription medicine in the incidence of an illness. The average age of respondents was 21 years. While average income was 208.69 Malawian Kwacha (MK), average household health expenditure was 4.55 MK. Further, less than 2% of respondents lived in communities with health facilities (including government, mission and private-for-profit clinics). Also, 27% of respondents assessed their health status as "somewhat better" than it was a year before the survey while 26% and 3% assessed their health as "much better" and "much worse" respectively.

**Table 2:** Descriptive statistics

Variable	Mean	Number (Percentage)
Non-prescription medicine use		1509 (56.49%)
Age	21	
Use of electricity		112 (4.17%)
**Education**		
None		2318 (86.27%)
Primary		364 (13.55%)
Secondary and above		5 (0.19%)
*Income (MK)	208.69	
Sex (Female)		1358 (50.54%)
Health Facility		40 (1.49%)
Toilet Facility		2254 (83.89
Bed Net		995 (37.04%)
Rural Residence		2454 (91.33%)
*Health Expenditure (MK)	4.5537	
**SAH**		
Much Better		655 (25.62%)
Somewhat Better		700 (27.38%)
About the same		669 (26.16%)
Somewhat worse		457 (17.87%)
Much worse		76 (2.97%)

*The exchange rate between Malawian Kwacha (MK) and United States Dollar in 2005 was US$1: 140 MK

### Determinants of non-prescription medicine use

Table 3 shows that individuals in communities which had health facilities were less likely to use non-prescription medicines. This relationship was significant at 1%.

**Table 3:** Probit regression analysis for determinants of non-prescription medicine use

Variable	Estimated coefficients	Standard errors
Health facility	-0.63336***	0.20894
Total health expenditure	0.01806**	0.00778
Use of electricity	-0.26491**	0.12249
Age square	-0.00009	0.00006
Age	0.00749*	0.00394
Household size	-0.00078	0.00908
Constant	0.02454	0.08688

Wald chi2	23.00***	
No of observations	2640	
Pseudo R2	0.0066	

Note: ***, **, * show significance at 1%, 5% and 10% respectively

The results also show that older individuals were more likely to use non-prescription medicine relative to the young. The relationship was significant at 10%.

The results further show that individuals living in households that spent more on health care were more likely to use non-prescription medicine. The relationship was significant at 5%. Finally, the use of electricity significantly relates to lower levels of non-prescription medicine use.

### Non-prescription medicine use versus self-assessed health

Table 4 shows that individuals who used non-prescription medicines were more likely to report lower categories of self-assessed health.

**Table 4:** Ordered probit regression analysis for self-assessed health and non-prescription medicine use

Variable	Estimated coefficients	Standard errors
Non-prescription medicine use	-0.07081*	0.04304
Use of electricity	-0.34715***	0.1067
Age	-0.00135	0.00117
**Education**		
Primary	0.10847*	0.06324
Secondary and above	0.1977	0.891
Log income	0.03039**	0.01401
Sex	0.02347	0.04253
Health facility	0.05702	0.18296
Toilet facility	-0.03026*	0.01625
Bed net	0.07709*	0.04501

Cut1	-1.98889***	0.07818
Cut2	-0.91146***	0.06593
Cut3	-0.91146***	0.06474
Cut4	0.56045***	0.06557

Wald chi2	203.50***	
No of observations	1186	
Pseudo R2	0.1734	

Note: ***, **, * show significance at 1%, 5% and 10% respectively

The results also show that individuals who had primary education were more likely to report some improvement in their health relative to individuals who had no formal education. The relationship was not significant for other levels of formal education.

Further, the results show that wealthier individuals were more likely to report "much better" health relative to poorer individuals. Also, the results show that individuals who used bed nets were more likely to report "much better" health as compared to those who did not use bed nets.

## Discussion

It has been established that lack of financial access to health care is a reason for non-prescription medicine use [4,21]. The current study, however, shows that geographical access to health facilities is another factor that influences the use of non-prescription medicines. This result is because geographical access to health facilities in a community makes it easy for sick individuals to seek treatment and prescription. Additionally, the availability of health facilities in a community reduces the cost of seeking treatment in terms of travel and waiting time and direct expenses. In this case, equity in access to treatment (including prescription) could be ensured with social health insurance schemes that lessen the financial burden of illnesses and facilitates expansion of health infrastructure.

Interestingly, the results suggest that households with higher health expenditure were more likely to use non-prescription medicines. This result contradicts the findings of Bush and Robin [22] whose evidence show that lower out-of-pocket costs for visits and prescribed medicines do not account for non-prescription medicine use. Much as this finding is surprising, the finding may be revealing the unwillingness of such households to spend any additional resources in acquiring prescription medicines. However, further evidence is required.

The findings again show that non-prescription medication use negatively influences individuals' perceived health as individuals who used such medicines were more likely to report lower levels of self-assessed health. This finding meets our expectation, as appropriate use of medicines plays a crucial role in improving people's perception of their health. However, Fillenbaum et al [12] found that poorer self-assessed health encourages the use of non-prescription medicines. Therefore, the current study and Fillenbaum et al's [12] study provide evidence that both self-assessed health and non-prescription medicine use influence each other.

## Conclusions

The study set out to examine the social and economic dimensions of the use of non-prescription medicines and to determine how non-prescription medicine use relates to self-assessed health of individuals. The results show that access to health facilities and household health expenditure are significant factors that influence the use of non-prescription medicine. Moreover, use of non-prescription medicine relates to lower levels of self-assessed health status of individuals in Malawi.

Notwithstanding, the relationship between non-prescription medicine use and self-assessed health need not be interpreted as a causal relationship, as the study did not seek to test a causality, but association between non-prescription medicines use and self-assessed health.

The findings imply that, in tackling the challenges posed by the use of non-prescription medicines in Malawi, the roles played by socio-economic characteristics of the population could provide useful information.

## Competing interests

The authors declare that they have no competing interests.

## Authors' contributions

JaN and JuN conceived the study, undertook analysis and drafted the manuscript. RM and TM contributed to the write up. All authors read and approved the final manuscript.
